# Multi-sector stakeholder consensus on tackling the complex health and social needs of the growing population of people leaving prison in older age

**DOI:** 10.1186/s40352-024-00271-y

**Published:** 2024-04-19

**Authors:** Ye In (Jane) Hwang, Stephen Hampton, Adrienne Lee Withall, Phillip Snoyman, Katrina Forsyth, Tony Butler

**Affiliations:** 1 School of Population Health, University of New South Wales, Sydney, NSW 2052 Australia; 2 Justice Health & Forensic Mental Health Network, Matraville, NSW 2036 Australia; 3 School of Psychology, University of New South Wales, Sydney, NSW 2052 Australia; 4 Corrective Services NSW, Haymarket, NSW 2000 Australia; 5 Health and Justice Research Network, University of Manchester, Manchester, M13 9PL UK; 6 Ageing Futures Institute, University of New South Wales, Sydney, NSW 2052 Australia

**Keywords:** Health equity, Social determinants of health, Recidivism, Population aging, Nominal group technique, Social services, Prisoners, Stakeholder cooperation; public health

## Abstract

**Background:**

As populations age globally, cooperation across multi-sector stakeholders is increasingly important to service older persons, particularly those with high and complex health and social needs. One such population is older people entering society after a period of incarceration in prison. The ‘ageing epidemic’ in prisons worldwide has caught the attention of researchers, governments and community organisations, who identify challenges in servicing this group as they re-enter the community. Challenges lie across multiple sectors, with inadequate support leading to dire consequences for public health, social welfare and recidivism. This is the first study to bring together multi-sector stakeholders from Australia to form recommendations for improving health and social outcomes for older people re-entering community after imprisonment.

**Results:**

A modified nominal group technique was used to produce recommendations from *N* = 15 key stakeholders across prison health, corrections, research, advocacy, aged care, community services, via online workshops. The importance and priority of these recommendations was validated by a broader sample of *N* = 44 stakeholders, using an online survey. Thirty-six recommendations for improving outcomes for this population were strongly supported. The key issues underlying the recommendations included: improved multi-stakeholder systems and services, targeted release preparation and practices that ensure continuity of care, advocacy-focused initiatives in the community, and extended funding for effective programs.

**Conclusions:**

There is consensus across stakeholders on ways forward, with intervention and policy updates required at the individual, systems and community levels. These recommendations entail two important findings about this population: (1) They are a high-needs, unique, and underserved group at risk of significant health and social inequity in the community, (2) Multi-sector stakeholder cooperation will be crucial to service this growing group.

**Supplementary Information:**

The online version contains supplementary material available at 10.1186/s40352-024-00271-y.

## Introduction

Worldwide, the number of older people in prisons is growing (Ginnivan et al., 2021; Merkt et al., 2020). Up to 1 in 4 people in prisons across the world are now older, and this proportion continues to grow (Australian Institute of Health & Welfare, 2019; Merkt et al., 2020). This ‘graying’ of prisoner populations worldwide emerged in literature in the early 2000s, with recognition of the phenomenon and its implications across multiple countries (Ahalt et al., 2013; Fazel et al., 2001; Ginnivan et al., 2021; Reimer, 2008). It has been referred to as a “crisis”, in recognition of the complex and multifaceted challenges it presents across justice, health and welfare sectors (Maschi et al., 2013; Scaggs & Bales, 2017). It is driven by factors including general population ageing, longer sentences, convictions for historical offences in later life, and trajectories of repeated imprisonment in certain high-risk groups (Howard & Corben, 2019; Luallen & Cutler, 2017; Roth, 2014; Scaggs & Bales, 2015). In Australia, the total prisoner population rose by 47% from 2009–2019, but the number of prisoners aged 50+ and 65+ rose by 81%, and 142%, respectively. This rate of growth exceeds that of the general community during the same period (25% and 81%, respectively).

Most people in prison will be released back into the community, and successful reintegration of this group is a challenge with older people in prison having poorer physical and mental health, and higher unmet health and social needs compared to both younger people in prison, and older people in the community (Australian Institute of Health & Welfare, 2019; Lee et al., 2019; Solares et al., 2020; Stevens et al., 2018). This challenge is exacerbated by prison systems that have been traditionally focused on security rather than care, and the stereotypical ‘young’ inmate (Davies, 2011; Hwang et al., 2021). These multiple vulnerabilities has resulted in consensus that in the prison context, people over 50 should be considered ‘older’ (Merkt et al., 2020). Resultantly, this group leave prison with the complex health and social service needs that are already associated with justice-involved populations, such as housing, employment and drug and alcohol services, but further compounded by this “accelerated ageing” or early onset of age-related conditions (Greene et al., 2018), as well as the social challenges and stigma associated with incarceration.

One review concluded that older people leaving prison suffer disproportionate challenges due to factors including: reduced social supports in the community, heightened health and mobility support needs, stigma, and difficulty adjusting psychologically following a life of institutionalisation (Davies, 2011). More recent literature confirms that these issues remain pertinent. For example, one study of older people (*N* = 101, Age 55+) in the U.S. found 46% of their sample had visited an emergency department within 6 months of release, with 21% having visited more than once (Humphreys et al., 2018). An Australian study reported that over half of its sample of released prisoners aged 45+ (*N* = 1,853) experienced discontinuity of their mental health care needs after release (Sodhi-Berry et al., 2015). There are also high suspected rates of cognitive impairment and dementia in older incarcerated adults, with staff and stakeholders voicing concern over inadequate services to meet these needs post-incarceration (Gaston, 2018; Stoliker et al., 2022). A recent cross-sectional study found that up to 45% of its sample of older adults at a forensic psychiatric facility in Canada (*N* = 29; Mean Age = 59.30) screened positive for dementia (Stoliker et al., 2022).

Qualitative work with this group and those involved in their care also highlights that reintegration difficulties for this group are manifold, including difficulties dealing with parole requirements, stigma, homelessness, job discrimination, technology use, essential life skills, seeking support services, rebuilding social connections, and managing multiple health conditions ( Jimenez et al., 2021; Lares & Montgomery, 2020; Wyse, 2018). Housing is a particularly challenging issue for this group in terms of their multifaceted health and care needs, coupled with a lack of resources. Stakeholders have urgently advised the increased availability of housing that is adequate to service their needs, as well as better assistance to access such options (Withall et al., 2022). People who have left prison in older age thus represent a highly medically and socially vulnerable population in the community, whose unmet needs carry costly consequences not just for the individual’s health and wellbeing, but also for health equity, social services, and recidivism.

In Australia, a growing body of literature recognises the rapid ageing of the prisoner population with qualitative studies, economic analyses, examination of offending data and commentaries conducted by academics and various government agencies all acknowledging this issue (Australian Institute of Health & Welfare, 2019; Ginnivan et al., 2021; Hagos et al., 2021; Howard & Corben, 2019; Hwang et al., 2021; Inspector of Custodial Services NSW, 2015; Office of the Inspector of Custodial Services WA, 2021; Simpson et al., 2017; Trotter & Baidawi, 2015). Whilst mostly focused on prisoner population ageing or prisoner health management in general, all emphasise the importance of appropriate post-release support for older people leaving prison. One qualitative study with correctional staff (*N* = 32) in Australia specifically focused on investigating the specific barriers and enablers to post-release reintegration in older people leaving prison, and identified that the challenges exist at not only the personal, but also the social, economic and organisational levels (Hagos, 2021).

Overall, the literature from both Australia and worldwide indicate that the concerted effort of multiple stakeholders across health, justice, research and social services both in prisons and in the community is needed to ensure successful reintegration of this group into the community (Hagos, 2021; Metzger et al., 2017; Schwartz et al., 2020). Metzger and colleagues’ 5-step COJENT framework (Criminal Justice Involved Older Adults in Need of Treatment) Initiative provides a useful framework to “determine the needs of criminal justice-involved older adults, assess the community’s relevant resources, identify knowledge and resource gaps, and to use this information to develop a stakeholder-driven action plan to address their needs” (Metzger et al., 2017, p. 2). The 5 steps include: (i) identifying multi-stakeholder perceptions of key issues, (ii) conducting community-based needs assessments, (iii) implementing quick-response interventions, (iv) holding public forums to engage stakeholders to prepare for action (v) consolidating the evidence and engaging “champions” to collaboratively develop and deliver an action plan.

This study aimed to bring together multiple stakeholders to form consensus on recommendations for improving health and social service delivery for older people leaving prison and to gain novel insights into key areas of need for this growing population. It also aimed to identify current strengths, resources and opportunities available to these stakeholders to begin working towards solutions. The study was designed in broad alignment with Step 1 of the COJENT framework and gathered useful information for informing Steps 2 and 3.

## Method

### Ethical approvals

This study was granted ethical approval from: The University of New South Wales Human Research EthicsCommittee [HC220042], Corrective Services NSW Ethics Committee [D2022/0294030], and the Justice Health and Forensic Mental Health Network of NSW Ethics Committee [G477/22].

### Design

**Fig. 1:**
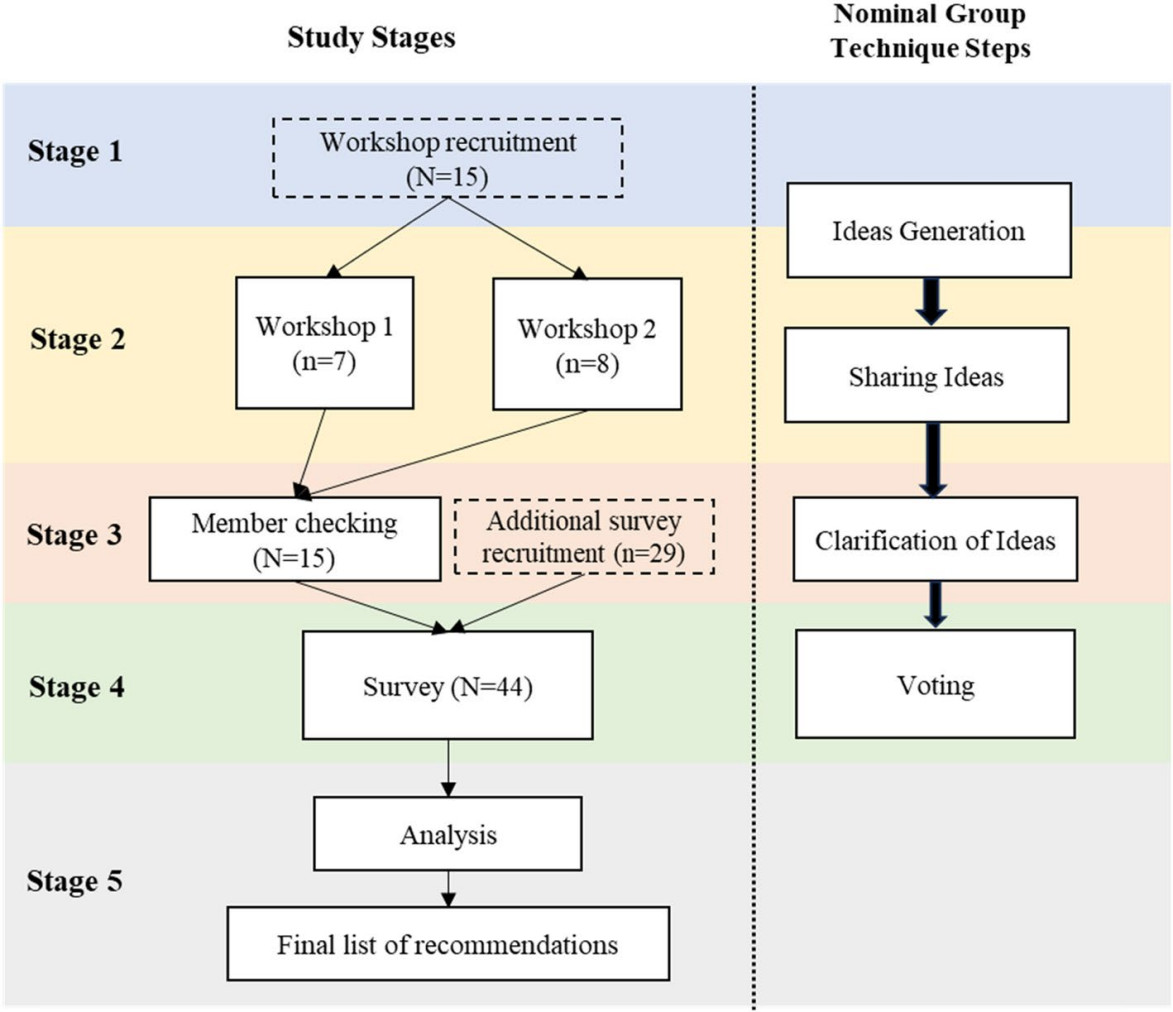
Stages of data collection and corresponding steps of nominal group technique

The overall aim of this study was to produce consensus on a list of recommendations for improving the health and social needs of older people leaving imprisonment. To do this, a modified version of the nominal group technique (McMillan et al., 2014, 2016) was used. The nominal group technique is a flexible, consensus-building method that allows for collaborative agreement to be reached whilst empowering each participant to contribute and have their ideas considered by others (McMillan et al., 2014). As a ‘face to face’ method, it fosters more immediate and dynamic exchange of ideas compared to the Delphi method, also reducing ambiguity or misunderstandings via direct interaction and feedback. It is also a more efficient method compared to the Delphi. The original design by Delbecq et al. (1975) involves four steps: *Silent Generation of Ideas* done individually, *Round Robin* sharing ideas as a group, *Clarification* of ideas, and *Voting* for ideas. These steps were undertaken across four stages in the present study: Pre-information worksheet, online workshops, member checking and online survey. See Fig. 1 for more detail.

### Stage 1 – workshop recruitment

#### Workshop sampling and recruitment

Workshop recruitment was targeted at six types of stakeholders: in-prison staff (health and custodial), community corrections, post-release transition support services, aged care providers, advocacy groups (older persons and justice-involved), and research academics. These stakeholders were chosen as those most knowledgeable and experienced in terms of the current practices surrounding the care and release of older people from prison in Australia. As the study aimed to recruit a relatively small sample of participants (*N* = 15) with specific expertise from a large pool of potential participants, purposive critical case sampling and snowball sampling was most suitable.

Inclusion criteria for the study included:

- At least 12 months professional experience dealing with older people leaving prison in Australia
- Age 18 + 

Exclusion criterion:

- Experience with older people leaving prison restricted to more than 5 years ago

For four of the stakeholder groups (post-release transition support services, aged care providers, advocacy groups and research academics), key organisations and individuals in Australia who would be best placed to comment on these matters were initially identified using the knowledge and expertise of the research team who have extensive research experience in this field. The organisations/groups were contacted via email or phone, either through publicly available contact details or existing professional contacts. A research team member explained the study, emailed study information, and sought assistance to identify potential participants for the study. A staff member within each organisation/group then agreed to send the relevant study materials to potential participants. Potential participants were individually invited to the study via an email invitation, which also included a copy of the Participant Information Statement and Consent Form, and a study flyer. Participants were able to express interest by contacting the research team via email or telephone. Upon expressing interest, the research team conducted an eligibility screen using the inclusion and exclusion criteria. An online version of the consent form was sent to eligible participants to be completed by clicking relevant checkboxes. Recruitment across groups occurred in parallel until a maximum of four participants from each stakeholder group was identified.

For the government stakeholder groups (in-prison staff and community corrections), ethics approval was only obtained for New South Wales, Australia. The research team similarly contacted the relevant contact within each department/agency, via publicly available details or existing contact details. The same process was undertaken as the other groups for identifying, contacting and gaining consent from potential participants.

#### Pre-workshop information

After the sample was recruited, the workshop date was set. Participants were then sent a pre-workshop information sheet, at least 1 week prior to their workshop date. This sheet clearly noted the aim of the workshop (to create a list of recommendations), and detail regarding what to expect during the workshop. This step was included to give participants ample time to consider the recommendations/ideas they would like to propose during the workshop.

### Stage 2 – workshops (ideas generation & sharing ideas)

Workshops were conducted online via Microsoft Teams and ran for 90 min. Two workshops were held (*n* = 7, *n* = 8) and participants attended one workshop depending on their availability. This meant we were able to elicit more and more, in-depth data from the fewer participants in each workshop and reduced the burden on participants to commit to a longer workshop.

Workshops were mediated by the first author and a research assistant. The main stages of the workshop included: participant introductions, a brief presentation on the current state of the literature, ‘warm up’ discussion question (“What are the key issues in health and social services for older people leaving prison?”). After this, participants were given 15 min to individually generate up to three recommendations each. Then, participants shared their recommendations with the group. The recommendations were scribed by the research assistant on a shared screen for all participants to see. Any overlapping recommendations were discussed and consolidated.

Fifty Australian dollars were available to participants as renumeration for their time. However, some participants who were employed by government agencies were unable to accept this payment. Video recordings of the workshop were downloaded at the end of each workshop and converted to audio-only files. These audio files were transcribed by the research assistant.

### Stage 3 – member checking (clarification of ideas)

After the workshops were complete, the first author and a research assistant worked together to consolidate the recommendations across both workshops. Potentially overlapping ideas were discussed and clarified by re-listening to each of the workshops. Member checking was then conducted by sending the consolidated list of recommendations, with rationale for all newly formed recommendations or those that were merged due to duplication, to all the workshop participants via email. Participants were given 2 weeks to respond to this email with feedback as to whether these recommendations reflected what was discussed in the workshop, and any suggestions for wording. Participants were asked to respond either way, to indicate whether they had any further feedback on this list. All participants responded to this email and one request was made for new wording of one recommendation. This requested change was accepted.

### Stage 4 – survey (voting)

The final list of recommendations was transferred onto an online survey on Qualtrics and sent back to the workshop participants (*N* = 15). Also, in addition to the workshop participants, we recruited a larger sample of relevant stakeholders to vote for the recommendations that were developed during the workshops (*N* = 35). Opening this final stage to a broader sample enabled us to gain wider endorsement of the final list of recommendations. This method of conducting a consensus workshop, then assessing representativeness against a wider sample has been used successfully in past research (Vella et al., 2000) to establish national priorities in critical care. The aim was to have a total of *N* = 50 participants to vote for the final list of recommendations. This sample included *N* = 15 from the original workshops, and an additional *N* = 35 participants.

Inclusion and exclusion criteria for the additional survey participants was the same as for the workshops. Recruitment processes were also similar. The same organisations/groups and individuals from the workshop recruitment processes were re-contacted and asked to disseminate study information emails and a copy of the consent form to potential participants. This email detailed the study and contained a link to an online form where potential participants read the online consent form. Interested participants could then complete an eligibility checklist via an online form, pertaining to the inclusion and exclusion criteria. Once deemed eligible, participants could proceed to complete the online consent form, and then complete the online survey.

The survey included a few questions asking the participant to detail their relevant expertise/interest in older people leaving prison. After this, participants were presented with the list of recommendations and asked to score each on a Likert scale, according to how important they believed the recommendation was (1 = not important, 9 = extremely important). They were then asked to select the top 7 that they believed were priority for implementation (in order). Participants could enter a draw to win an online gift card 100AUD in value.

### Stage 5 – analysis and final list of recommendations

The survey was closed after 3 months. The researchers manually reviewed the screening questions to ensure only eligible participants’ responses were included for analysis. For analysis, we referred to methods by McMillan and colleagues regarding analysis across multiple groups in nominal group technique (2014). Ranking scores were imported into Microsoft Excel. All items were then ranked according to medians. Medians of 7–9 were defined as ‘strong support’, 4–6 as moderate, and 1–3 as weak. The level of support for each item was assessed by the median score, and the level of agreement within each group of similar stakeholders was assessed using the mean absolute deviation from the median. The mean absolute deviation is an indication of variability.

Scores for the top 7 priorities for implementation were calculated using a weighted sum approach. Items ranked higher were given a higher weighted score, i.e., (ranked 1^st^ = 7 points, 2^nd^ = 6 points, 3^rd^ = 5 points, 4^th^ = 4, 5^th^ = 3, 6^th^ = 2 points, 7^th^ = 1 point). A final score for each item was calculated based on the sum of these weighted values.

## Results

### Workshops

Two workshops were conducted in June 2022, and *N* = 15 participants took part (Table 1). Whilst we cannot detail the specific experience represented by each of the stakeholders for privacy concerns related to small sample size, the critical case sampling employed ensured that those with strong expertise on the topic were identified to participate in the study. Each participant often noted having numerous years of experience across multiple roles within justice health, corrections and transition support across Australia. The academics all had a combination of publishing papers, acquiring research funding and running research projects on the topic of older incarcerated people in Australia. All stakeholders were from two states: New South Wales and Victoria.

**Table 1:** Nature of participants in consensus workshops

**Stakeholder type**	**n**
Prison health provider	3
Corrective services (custody and community)	3
Post-release transition support services	4
Advocacy and community groups	2
Researcher	3

The workshops resulted in a final list of 37 recommendations that could be divided into nine categories. Brief category descriptions are provided below (see also Additional file 1).

A. *Working Together, Better (7 recommendations)*

Systems and resources that enable different stakeholders to work more effectively in collaboration with one another, allowing smooth transition into the community.

B. *Age-appropriate Care in Prison (3 recommendations)*

Health-focused practices to ensure age-related health of each person is managed and assessed, ready for handover to health and aged care services in the community.

C. *Preparing the Individual (5 recommendations)*

Courses and programs during incarceration that increase a person’s ability to undertake key activities in the community (e.g., cooking, using technology).

D. *Intense, Attentive and Individualised Release Planning (4 recommendations)*

Release planning that begins much earlier during incarceration, developed with and for the individual, and is attentive to their immediate needs after release.

E. *Doing Things Differently (6 recommendations)*

Calls to revise existing custodial and parole policies to better capture the unique needs of older people leaving prison.

F. *Community Programs and Support (3 recommendations)*

Initiatives in the community to help prison leavers adjust and find support in several areas.

G. *Advocacy, Awareness and Stigma (5 recommendations)*

Increasing awareness and decreasing stigma regarding older people leaving prison for the general public, aged care and health services. Also, advocacy initiatives directed at the government.

H. *Specific Sub-populations (2 recommendations)*

Filling service and policy gaps for women and Aboriginal or Torres Strait Islander people.

I. *Funding (2 recommendations)*

Increased, consistent and more sustainable funding to ensure effective programs can be tested and continue to be delivered.

### Survey

In total, *N* = 44 eligible participants completed the online survey over 3 months (Table 2). All workshop participants also completed the survey.

**Table 2:** Nature of online survey participants

**Stakeholder type**	**n**
Prison health provider	15
Corrective services (custody and community)	14
Post-release transition support services	5
Advocacy and community groups	2
Researcher	5
Other government agency	2

#### Importance and level of agreement

All respondents (*N* = 44) ranked the 37 recommendations in terms of importance. The results are displayed in Table 3. Each item is presented in order of their median importance score and level of agreement (calculated as the mean absolute deviation). The mean absolute deviation illustrates how much agreement there is between respondents in their rating of each item. A smaller mean absolute deviation indicates higher agreement between respondents. All but one of the recommendations were strongly supported, indicated by a median score of 7–9.

**Table 3:** Final recommendations in order of importance (median score) and level of agreement (mean absolute deviation)

Rank	Item	Importance (Median)	Level of agreement (Mean Absolute Deviation)
1	Immediate health needs on release should be taken care of (Uninterrupted access to mobility equipment, sufficient prescription medication to outlast any service delays or public holidays)	9	0.39
2	Introduce health assessments in prison that are aligned with Commonwealth funded aged care services, that also include a risk assessment component	9	0.44
3	Cognitive function and dementia assessment/diagnosis should be available to all older people in prison	9	0.61
4	Clearer responsibilities and roles on the part of community services (e.g., disability, aged care) to remove the risk of this group falling between the gaps	9	0.66
5	Improved systems for information sharing, administration and communication between stakeholders and services	9	0.72
6	Longer and consistent funding to allow programs to be piloted, evaluated and implemented	9	0.76
6	Sustainable funding models for programs that are found to be effective	9	0.76
8	Establishment of an independent reintegration team to liaise with all the different groups and services involved in release planning	9	0.79
9	Transition planning should occur as early as possible during a person’s time in custody (ideally at least 3–6 months prior to release)	9	0.83
9	Fill service gaps for First Nations prison leavers who have unique cultural and health needs	9	0.83
11	Increased housing options specifically for older people who are leaving prison, especially those convicted of sex crimes	9	0.85
12	Seamless transition between state or commonwealth services and in-prison services for people entering and leaving prison (ie going from a Medicare to Justice Health environment, and leaving again)	9	1.05
13	Fill service gaps for women leaving prison in older age	9	1.07
14	Existing transition programs should review their criteria to allow increased eligibility of older people who may not be ‘high risk’	9	1.12
14	Increased cooperation from Local Health Districts for release planning^a^	9	1.12
16	Parole boards should reconsider programs that are not suited for older people due to issues such as cognitive ability	9	1.27
17	Preventative functional maintenance programs are needed to prevent deterioration during incarceration	8	0.80
18	Intense, person-centered case management approach developed with the input of the individual	8	0.97
19	Government funded, centralised transition support and advocacy roles that bridge pockets of practice across areas	8	1
20	Increased use of diversion policies for older people who could be better housed/rehabilitated elsewhere	8	1.02
21	Activities in the community to help make new social connections	8	1.05
22	Increased independence and responsibility during incarcerated life to emulate more real-world conditions (e.g., responsibility for meals)	8	1.15
23	Life skills courses to prepare for release that is focused on daily living (e.g., cooking, transport) and accessing services (e.g., going to the bank)	8	1.17
24	Prison leavers should have a physical transition support “package” in hand (including e.g., key contacts for local services, tips, to do lists, medical records and identification)	8	1.17
25	Programs to increase self-efficacy and agency in older people leaving prison	8	1.20
26	Digital literacy/ technology readiness programs (e.g., smartphones, internet, accessing services online) for older people	8	1.22
26	Increased education for nursing homes and aged care staff to reduce stigma and increase confidence	8	1.22
28	A review of medical parole policies and their apparent underutilisation	8	1.27
29	Older people leaving prison should be deemed a priority population for the Commonwealth funded Care Finders initiative to help access aged care services (includes a workforce of First Nations facilitators)^b^	8	1.37
30	Release planning should occur regardless of risk level	8	1.41
31	Release ‘practice’ via excursions, or immersive experiences (e.g., videos, role play, virtual reality) to increase familiarity with post-release life	8	1.44
32	A trauma-informed care framework should be adopted by all stakeholders	8	1.46
33	Initiatives to increase public awareness of the existence of this population and the societal economic and human rights implications	8	1.66
34	Initiatives to address stigma in the general public towards prison leavers, especially against those convicted of sex crimes	8	1.95
35	A state/national forum for stakeholders to share experiences and plan to work together better	7	1.47
36	Peer mentoring by someone with lived experience, involving both counselling and moral support	7	1.66
37	Increased involvement from religious groups in the community to meet spiritual and social needs	6	1.95

^a^Local Health Districts exist only in New South Wales. The state is divided into 15 Local Health Districts with the objective of making decisions and delivering healthcare at a local level

^b^The Care Finders is a free, Commonwealth (national) government-funded service to assist vulnerable older peoplefind aged care and other relevant support

#### Priorities for implementation by stakeholder group

After ranking the importance of each recommendation, participants chose the Top 7 recommendations that they believed were priorities for implementation (Table 4). The priority ranks and total score for each recommendation are presented first for the entire sample (*N* = 44), followed by a breakdown by stakeholder type. Groups with small sample sizes (*N* < 5; transition support workers, advocacy groups and other government stakeholders) were collapsed into one.

**Table 4:** Priority rank and score for all recommendations by stakeholder type

Item	Priority rank (score)				
	All groups (*N* = 44)	Prison health (*N* = 15)	Corrective Services (*N* = 14)	Transition support & other (*N* = 9)	Researcher (*N* = 6)
Establishment of an independent reintegration team to liaise with all the different groups and services involved in release planning	1 (145)	1 (64)	1 (27)	1 (29)	10 (5)
Improved systems for information sharing, administration and communication between stakeholders and services	2 (108)	2 (53)	11 (16)	5 (21)	2 (18)
Transition planning should occur as early as possible during a person’s time in custody (ideally at least 3–6 months prior to release)	3 (89)	6 (26)	5 (22)	6 (20)	1 (21)
Immediate health needs upon release should be taken care of (Uninterrupted access to mobility equipment, sufficient prescription medication to outlast any service delays or public holidays)	4 (79)	3 (41)	12 (15)	8 (11)	3 (15)
Digital literacy/ technology readiness programs (e.g., smartphones, internet, accessing services online) for older people	5 (76)	5 (27)	6 (21)	2 (23)	6 (11)
Cognitive function and dementia assessment/diagnosis should be available to all older people in prison	6 (72)	14 (11)	2 (35)	2 (23)	10 (5)
Intense, person-centered case management approach developed with the input of the individual	7 (69)	8 (22)	15 (10)	2 (23)	4 (14)
Life skills courses to prepare for release that is focused on daily living (e.g., cooking, transport) and accessing services (e.g., going to the bank)	8 (67)	4 (40)	4 (27)	-	-
Increased housing options specifically for older people who are leaving prison, especially those convicted of sex crimes	9 (64)	16 (6)	3 (32)	7 (12)	4 (14)
Prison leavers should have a physical transition support “package” in hand (including e.g., key contacts for local services, tips, to do lists, medical records and identification)	10 (53)	7 (23)	9 (18)	8 (11)	23 (1)
Increased independence and responsibility during incarcerated life to emulate more real-world conditions (e.g., responsibility for meals)	11 (42)	9 (21)	12 (15)	15 (6)	-
Release ‘practice’ via excursions, or immersive experiences (e.g., videos, role play, virtual reality) to increase familiarity with post-release life	12 (34)	13 (12)	10 (17)	11 (10)	-
Programs to increase self-efficacy and agency in older people leaving prison	13 (33)	10 (19)	22 (7)	8 (11)	-
Older people leaving prison should be deemed a priority population for the Commonwealth funded Care Finders initiative to help access aged care services	13 (33)	16 (6)	7 (19)	-	7 (8)
Increased use of diversion policies for older people who could be better housed/rehabilitated elsewhere	15 (29)	11 (15)	20 (9)	20 (3)	20 (2)
Introduce health assessments in prison that are aligned with Commonwealth funded aged care services, that also include a risk assessment component	16 (28)	12 (14)	-	11 (10)	10 (5)
Initiatives to address stigma in the general public towards prison leavers, especially against those convicted of sex crimes	17 (27)	19 (1)	14 (11)	11 (10)	10 (5)
Sustainable funding models for programs that are found to be effective	18 (26)	-	15 (10)	11 (10)	7 (8)
Fill service gaps for First Nations prison leavers who have unique cultural and health needs	19 (19)	-	7 (19)	-	-
A trauma-informed care framework should be adopted by all stakeholders	20 (17)	-	15 (10)	16 (4)	17 (3)
Preventative functional maintenance programs are needed to prevent deterioration during incarceration	21 (16)	-	15 (10)	22 (2)	15 (4)
Seamless transition between state or commonwealth services and in-prison services for people entering and leaving prison (ie going from a Medicare to Justice Health environment, and leaving again)	22 (14)	18 (5)	25 (3)	16 (4)	20 (2)
Government funded, centralised transition support and advocacy roles that bridge pockets of practice across areas	23 (13)	-	15 (10)	-	17 (3)
Clearer responsibilities and roles on the part of community services (e.g., disability, aged care) to remove the risk of this group falling between the gaps	24 (12)	-	21 (8)	-	17 (3)
Increased education for nursing homes and aged care staff to reduce stigma and increase confidence	24 (12)	-	15 (10)	-	20 (2)
Longer and consistent funding to allow programs to be piloted, evaluated and implemented	26 (10)	-	28 (1)	20 (3)	9 (6)
A state/national forum for stakeholders to share experiences and plan to work together better	27 (7)	-	27 (2)	-	10 (5)
Release planning should occur regardless of risk level	27 (7)	15 (7)	-	-	-
Increased cooperation from Local Health Districts for release planning	27 (7)	-	22 (7)	-	-
Peer mentoring by someone with lived experience, involving both counselling and moral support	30 (5)	19 (1)	-	16 (4)	-
Parole boards should reconsider programs that are not suited for older people due to issues such as cognitive ability	31 (4)	-	-	16 (4)	-
Initiatives to increase public awareness of the existence of this population and the societal economic and human rights implications	31 (4)	-	-	-	15 (4)
Activities in the community to help make new social connection	33 (3)	-	25 (3)	-	-
A review of medical parole policies and their apparent underutilisation	34 (2)	-	-	22 (2)	-
Existing transition programs should review their criteria to allow increased eligibility of older people who may not be ‘high risk’	35 (1)	19 (1)	-	-	-
Increased involvement from religious groups in the community to meet spiritual and social needs	-	-	-	-	-
Fill service gaps for women leaving prison in older age	-	-	-	-	-

The top five recommendations across all groups were:

1. Establishment of an independent reintegration team to liaise with all the different groups and services involved in release planning
2. Improved systems for information sharing, administration and communication between stakeholders and services
3. Transition planning should occur as early as possible during a person’s time in custody (ideally at least 3–6 months prior to release)
4. Immediate health needs upon release should be taken care of (Uninterrupted access to mobility equipment, sufficient prescription medication to outlast any service delays or public holidays)
5. Digital literacy/ technology readiness programs (e.g., smartphones, internet, accessing services online) for older people

The top priority was common across all groups except for the researcher group. Whilst there was some spread evident in the top priorities across stakeholder groups, there was a general tendency for the higher-ranking priorities to be common across groups.

## Discussion

### Overview of findings

This deliberative study produced 36 recommendations for improving the health and social outcomes of older people leaving prison with strong support from multiple stakeholders, and one recommendation with moderate support. Overall, the underlying issues were aligned with those identified in other countries such as the United States and United Kingdom (Metzger et al., 2017; O’Hara et al., 2015), with some more specific suggestions relevant to the Australian context. The findings confirm the recommendations made in a previous Australian roundtable on supporting people released from prison (i.e., referral practices, health needs, housing and funding stability) (Schwartz et al., 2020), whilst extending these to consider multistakeholder cooperation and issues that are pertinent to this age group. Overall, the study findings may be synthesised into two important lessons regarding the growth of this population, that are discussed further in proceeding sections:

1. They are a high-needs, unique, and underserved group at risk of significant health and social inequity in the community
2. Multi-sector stakeholder cooperation will be crucial to service this growing group

The ranking of priorities for implementation should be interpreted with care due to the small number of respondents in certain stakeholder groups. All but one of the recommendations were voted with strong support. Thus, the overall order of the priorities in Table 4 should not be interpreted as an indication of which recommendations are more important than others. Rather, the order reflects a combination of urgency for implementation, as well the likelihood of multistakeholder support. The differences in ranking between stakeholder groups reflects that each group has experienced varied exposure to different parts and consequences of the release process and may indicate which groups may be poised to provide more (or less) support or drive certain recommendations.

### Key issues and implications

#### High-needs, unique and underserved group

These findings identify that older people leaving prison have high and complex needs that are not well serviced with existing practices and resources. Whilst the areas of concern are similar to that of younger people leaving prison, there are unique and escalated needs for this age group, which stem from: more complex and heightened health needs at release, higher risk of homelessness, often longer experiences of institutionalisation, lack of age-appropriate care and release planning whilst incarcerated, and a lack of advocacy. Five of the recommendations thus call for changes in practices for services dealing with this population during and after release (Additional file 1, Category E). These suggestions touch on three specific areas including trauma-informed care, using ‘needs’ rather than ‘risk’ as the determining factor for service allocation in this group, and alternative housing (outside of prison in other secure settings) for those who are unwell and a low risk to the community.

A repeated concern is the risk of this population falling through service gaps in the community. According to these findings, these gaps emerge because of the multifaceted nature of the older person’s needs, the lack of support and advocacy to bridge these needs, a lack of continuity between in prison and community services, and unclear roles on the part of each ‘receiving’ service in the community (e.g., aged care, disability services) in meeting these needs. Older women and Aboriginal or Torres Strait Islander peoples were identified as particularly vulnerable. Housing is arguably the most basic service need to be fulfilled before others can also be met. Housing options are urgently needed, with the right care and social provisions are needed to adequately service the high and multifaceted needs of this group. This is in line with existing literature that recognises these groups experience additional health needs that intersect with old age and imprisonment, that often remain unmet after release (Abbott et al., 2017; Day & Tamatea, 2019; Handtke et al., 2015).

Also evident is apparent inadequacy in release planning for this age group. Stakeholders supported the need for individuals to access a broader range of health assessments and support resources to take care of their health and service needs after release. This aligns with evidence indicating that this group has high and immediate needs after release due to multiple health conditions, lost social connections, housing and financial instability (Australian Institute of Health & Welfare, 2019; Lares & Montgomery, 2020), as well as international studies indicating that release planning for this age group is both lacking and entails significant negative consequences (Forsyth et al., 2015; Hagos, 2021; O’Hara et al., 2015).

Importantly, additional resources are needed to adequately service this growing group. Two of the recommendations (including the top-ranked priority) called for independent, government-funded roles or services to liaise between the multiple stakeholder groups, and to provide advocacy for their needs. This reflects several underlying issues. That is, the needs of older people leaving prison span multiple services and sectors with significant work involved in liaising with each of these. Also, these tasks do not naturally fit within the remit of any existing staff roles whether in health, community services, or correctional services. Finally, existing staff are not adequately resourced to complete these tasks. Thus, there is a need for an independent and dedicated role or service to be created.

This ambiguity of staff roles arising from the complex, interconnected health, social and criminogenic needs of older people leaving prison has been raised in previous research (Hagos et al., 2021). The preference for an independent role may reflect a lack of resources on the part of existing staff to complete these tasks, and/or the belief that no existing service is currently suitable to take on this role. In either case, with the rise in this population a new area of need has arisen that requires resources to adequately service. A review of the additional tasks arising at the intersection of these multiple services, and the unique expertise that different stakeholders may already have, will provide direction on how to begin servicing this need. Economic evaluations of the potential benefits of such a role in improving public health outcomes, reducing unemployment and recidivism, can assist in this case.

An important hurdle identified in this work for servicing needs and allocation of resources, is the stigma experienced from services and people in the community. The recommendations call for increased advocacy, awareness and reduced stigma, particularly in securing aged care and housing. Seeking stable housing is a challenge for people leaving prison at all ages (Schwartz et al., 2020). Additional complexity is present where aged care is needed in an already-limited housing landscape. Advocacy and stigma-reducing initiatives are particularly relevant for older people leaving prison, as they are more likely to have been imprisoned for more serious offences that carry long sentences. Discussions around this recommendation pointed to the existence of both formal (practice) and attitudinal barriers that aged care providers and the general community in allocating public resources to previously incarcerated people. The precise nature of such barriers should be explored further in qualitative work and policy reviews and followed by appropriate responses to address these.

#### The need for multistakeholder cooperation

Many highly ranked recommendations confirm the need for improved ways of working together for the multiple stakeholders involved in reintegration of older people leaving prison. This is aligned with the purpose of the COJENT framework (Metzger et al., 2017) which underlies this work, and confirms existing findings regarding disparate and siloed ways of working even between stakeholders within the prison context, such as prison health services and corrective services (e.g., Hagos, 2021). This also aligns with the World Health Organization (WHO) Prison Health Framework Priority to “Conduct intersectoral work for better performance and outcomes” (World Health Organization, 2021, p. 6).

**Fig. 2:**
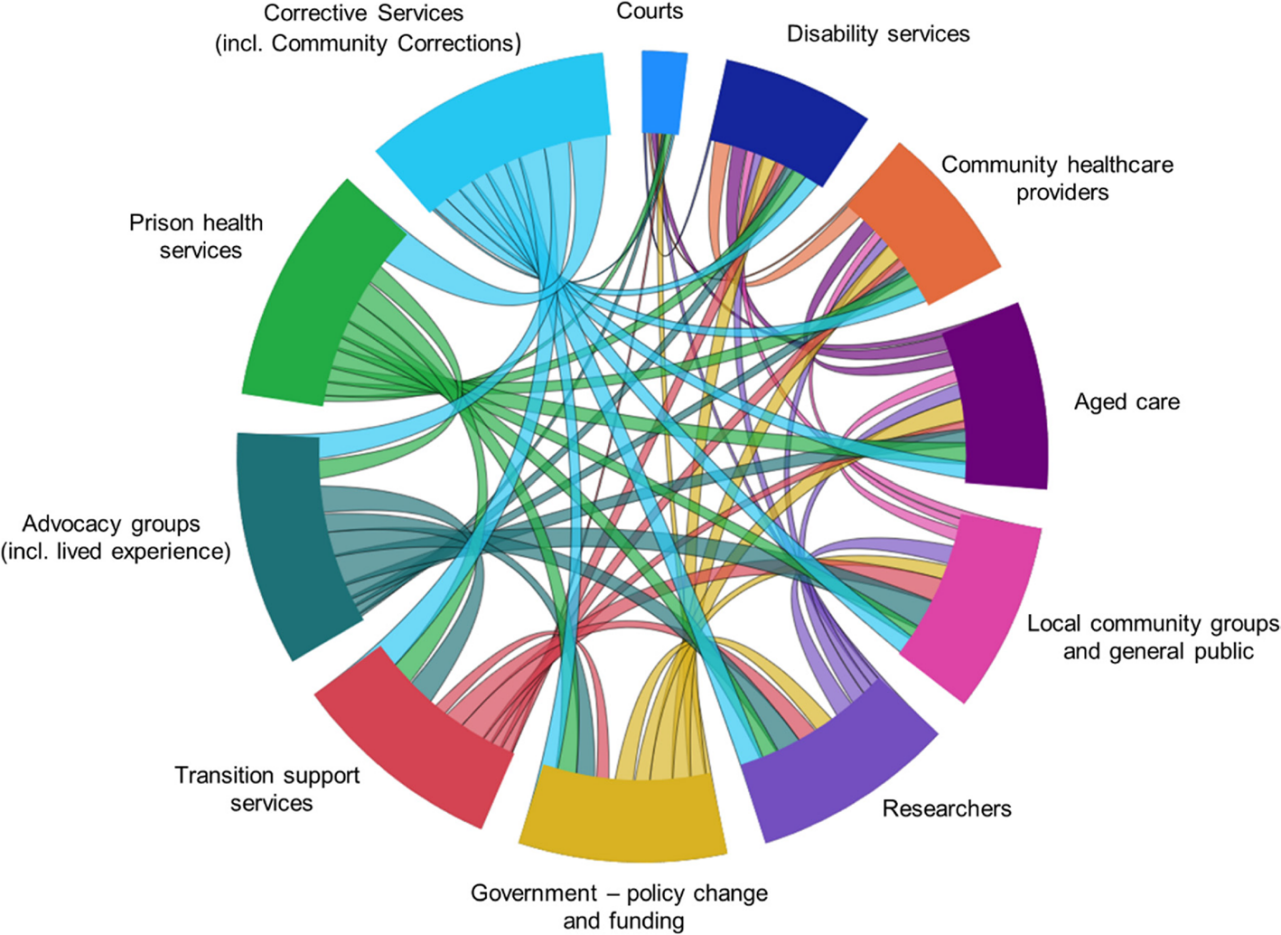
Chord diagram representing shared delivery of recommendations produced in this study. Larger chord volume indicates a higher number of shared recommendations with other stakeholders

The need for multistakeholder cooperation is illustrated well in Fig. 2, where we mapped each recommendation to each stakeholder that will be primarily involved in implementing that recommendation. The volume of each chord connecting stakeholders provides an indication of how many recommendations they have in common. Inevitably, the bulk of the work will need to occur with the cooperation of corrective services (both correctional facilities and community corrections). This work urges the establishment of new or closer collaborations between corrective services and many other stakeholders in the community, with long term benefits for all stakeholders involved both in terms of resource use and more effective servicing of the health and social needs of this population.

Throughout the recommendations there is a clear emphasis on the importance of preparing an older person for post-prison life whilst they are still in custody (Additional file 1; Categories B-D). Psychological and practical buffers are needed to ease transition between a highly regimented and uniquely restricted life in custody, and independent life in the community. Such programs are unlikely to be feasibly implemented through the expertise and resources of corrective services alone. Rather, ‘lowering the walls’ to permit older people in prison to access varying programs offered by community-based organisations prior to their release will be beneficial. In many ways this population are among the most marginalised sectors of society and have been identified as a primary target for public health interventions (Kinner & Wang, 2014; The Lancet, 2021). Existing programs for older people in the community in key areas such as health literacy and digital literacy can be brought to this population, to provide benefits not only for community reintegration but for public health and social welfare overall.

These findings show that stakeholders strongly support initiatives that work towards the ideal of reduced gaps between prison and community services. The key to achieving this, whilst also addressing the risk of falling between service gaps, appears to be in encouraging continuity of care via integrated service delivery across prison and the community. Unequal and discontinued care coverage both during incarceration and after release is a global issue (Winkelman et al., 2022), and the current findings arise in the context of sustained efforts by Australian stakeholders to lobby for the extension of the universal healthcare insurance scheme (Medicare) to people in prison with clear human rights, economic and public health benefits (Cumming et al., 2018). To our knowledge, no models of integrated service delivery between prison, health and community services have been developed or tested. Such efforts align with the principle of ‘equivalence of care’, an obligation for prison health services in Australia as well as ‘universal health coverage’ an identified priority by the World Health Organization and mandated by the UN Nelson Mandela Rules (Jotterand & Wangmo, 2014; World Health Organization, 2021, p. 6).

A strongly supported recommendation was for shared information, communication and administrative systems that are accessible across multiple organisations. This will be the capstone recommendation for enabling better working relationships among stakeholders. It also aligns with the WHO Prison Health Framework Priority to “Strengthen prison information systems to enhance surveillance and response capacity” (World Health Organization, 2021, p. 6), with outdated information systems identified as an ‘avoidable barrier’ to improved health outcomes in those who experience incarceration (Winkelman et al., 2022). The creation of shared systems across health, social service and correctional services across both prisons and the community is a complex task, involving cooperation between research, government and industry to ensure the solution is evidence-based and effective. Some foreseeable challenges will include differing levels of digital readiness at both systems and workforce levels, reconciling competing interests, addressing privacy or security concerns, and involving end users in a meaningful way to the design process. However, there is a clear need for a solution to address the obstacles experienced by stakeholders in accessing important health and personal information in a timely fashion.

### Strengths and limitations

A strength of this study was in bringing together the perspectives of multiple stakeholders both in prisons and the community, to advance an agenda for policy, practice and research. To our knowledge it is the first study that has done this in Australia. In the final sample (*N* = 44), there was strong representation from the prison health and corrective services stakeholder groups, albeit with less representation from those in research, advocacy, community services and aged care. This was due to a comparatively smaller pool of individuals with the level of expertise required to participate. In particular, only a small proportion of the sample represented aged care, advocacy and research. Despite this, aged care and advocacy issues were well-represented in the final recommendations. Overall, we believe the expertise of the selected participants in the workshops was strong, and a crucial mass of expertise on this population was achieved. Notwithstanding these limitations, the workshops that informed the recommendations were comprehensive, and resultant issues were all subsequently strongly endorsed by the wider sample. In moving forward, active involvement from appropriate stakeholders from aged care, advocacy and research in designing and implementing solutions will be important.

Due to ethical and administrative barriers and resource limitations, the study sample were predominantly those currently affiliated with two Australian jurisdictions (New South Wales and Victoria). Whilst some limit on generalisability will exist, the sample nevertheless provided strong representation of the key issues. New South Wales and Victoria represent the first and third largest population of prisoners cross the eight states and territories respectively, managing almost half (47.4%) of prisoners in Australia (ABS, 2021). Many workshop participants also (*N* = 15) reported extensive experience and knowledge that spans multiple jurisdictions in Australia. Whilst local solutions will be needed, the arising issues appear to have universal applicability and reflect concerns in existing literature both in Australia and worldwide.

An important gap in this work is the views of those with lived experience of leaving prison in older age. Research is needed to capture both their experiences of navigating post-release life as well as their priorities and recommendations for improving outcomes during this time. This group should also be centrally involved in designing and implementing solutions.

### Conclusions

Multiple stakeholders across health, justice, research, and social services have common interests and a clear need to work together to ensure positive outcomes for the growing and vulnerable population of older people leaving prison. Resources, programs and policy change is needed at multiple levels, including the individual, organisational, governmental and wider society. There are similarities in the needs and that are documented for younger people leaving prison, and thus it is likely that implementing these recommendations will also translate to benefits for prison leavers of all ages. Whilst not all recommendations will be feasible to implement, similar solutions that meet these key areas of need should be pursued. Next steps in this work will involve the continued application of the COJENT framework to achieve this, i.e., (ii) conducting community-based needs assessments, (iii) implementing quick-response interventions, (iv) holding public forums to engage stakeholders to prepare for action (v) consolidating the evidence and engaging “champions” to collaboratively develop and deliver an action plan (Metzger et al., 2017). This work should also progress the development of a national strategy for improving release outcomes for this population, providing a practical imperative for further work.

### Supplementary Information

**Supplementary Material 1.**

## Data Availability

The datasets generated and/or analysed during the current study are not publicly available because ethical approval was not given to share this data publicly, for reasons including potential re-identifiability of qualitative responses to the interview questions. Consent for data sharing was not granted by study participants.
