# Supplementary material for: Multi-sector stakeholder consensus on tackling the complex health and social needs of the growing population of people leaving prison in older age
Source: Health Justice. 2024 Apr 19;12:17. doi: 10.1186/s40352-024-00271-y (PMC11027373; doi:10.1186/s40352-024-00271-y)
Supplement: Supplementary file 1 — Supplementary Material 1. [file 40352_2024_271_MOESM1_ESM.docx]

# Additional File 1

*List of final consensus recommendations by category*

Category A. Working Together, Better

1. Improved systems for information sharing, administration and communication between stakeholders and services
2. Seamless transition between state or commonwealth services and in-prison services for people entering and leaving prison (ie going from a Medicare to Justice Health environment, and leaving again)
3. Clearer responsibilities and roles on the part of community services (e.g., disability, aged care) to remove the risk of this group falling between the gaps
4. Increased cooperation from Local Health Districts for release planning
5. A state/national forum for stakeholders to share experiences and plan to work together better
6. Establishment of an independent reintegration team to liaise with all the different groups and services involved in release planning
7. Government funded, centralised transition support and advocacy roles that bridge pockets of practice across areas

Category B. Age-appropriate Care in Prison

1. Cognitive function and dementia assessment/diagnosis should be available to all older people in prison
2. Preventative functional maintenance programs are needed to prevent deterioration during incarceration
3. Introduce health assessments in prison that are aligned with Commonwealth funded aged care services, that also include a risk assessment component

Category C. Preparing the Individual

1. Life skills courses to prepare for release that is focused on daily living (e.g., cooking, transport) and accessing services (e.g., going to the bank)
2. Digital literacy/ technology readiness programs (e.g., smartphones, internet, accessing services online) for older people
3. Increased independence and responsibility during incarcerated life to emulate more real-world conditions (e.g., responsibility for meals)
4. Programs to increase self-efficacy and agency in older people leaving prison
5. Release 'practice' via excursions, or immersive experiences (e.g., videos, role play, virtual reality) to increase familiarity with post-release life

Category D. Intense, Attentive and Individualised Release Planning

1. Transition planning should occur as early as possible during a person’s time in custody (ideally at least 3-6 months prior to release)
2. Intense, person-centered case management approach developed with the input of the individual
3. Prison leavers should have a physical transition support “package” in hand (including e.g., key contacts for local services, tips, to do lists, medical records and identification)
4. Immediate health needs upon release should be taken care of (Uninterrupted access to mobility equipment, sufficient prescription medication to outlast any service delays or public holidays)

Category E. Doing Things Differently

1. A trauma-informed care framework should be adopted by all stakeholders
2. Parole boards should reconsider programs that are not suited for older people due to issues such as cognitive ability
3. Release planning should occur regardless of risk level
4. Existing transition programs should review their criteria to allow increased eligibility of older people who may not be ‘high risk’
5. Increased use of diversion policies for older people who could be better housed/rehabilitated elsewhere
6. A review of medical parole policies and their apparent underutilisation

Category F. Community Programs and Support

1. Peer mentoring by someone with lived experience, involving both counselling and moral support
2. Activities in the community to help make new social connections
3. Increased involvement from religious groups in the community to meet spiritual and social needs

Category G. Advocacy, Awareness and Stigma

1. Initiatives to increase public awareness of the existence of this population and the societal economic and human rights implications
2. Increased education for nursing homes and aged care staff to reduce stigma and increase confidence
3. Initiatives to address stigma in the general public towards prison leavers, especially against those convicted of sex crimes
4. Older prison leavers should be deemed a priority population for the Commonwealth funded Care Finders initiative to help access aged care services (includes a workforce of First Nations facilitators)
5. Increased housing options specifically for older people who are leaving prison, especially those convicted of sex crimes

Category H. Specific Sub-populations

1. Fill service gaps for First Nations prison leavers who have unique cultural and health needs
2. Fill service gaps for women leaving prison in older age

Category I. Funding

1. Longer and consistent funding to allow programs to be piloted, evaluated and implemented
2. Sustainable funding models for programs that are found to be effective
